# Assessment of Confidence in Communication Skills and Its Practice With Patients Among Medical Professionals in Trichy, India

**DOI:** 10.7759/cureus.35818

**Published:** 2023-03-06

**Authors:** Puhalenthi K, Prasad S, Chandrababu C, Roshini S

**Affiliations:** 1 Community Medicine, K.A.P. (Krishnan Arumugam Periyannan) Viswanatham Government Medical College, Trichy, IND; 2 Paediatrics, Trichy SRM (Sri Ramasamy Memorial) Medical College Hospital and Research centre, Trichy, IND

**Keywords:** health communication, interpersonal communication skills (ics), professional communication, patient-doctor communication, medical education

## Abstract

Introduction

One of the most fundamental aspects of medicine is the doctor-patient relationship. Many factors influenced this link, including socio-cultural patterns, economic levels, political systems, and health systems. In India, there is an increase in violence toward physicians. Almost every now and then, the newspaper comes with headlines about a doctor being abused by patients or their families. Concerns regarding the absence of good doctor-patient communication among Indian doctors prompted an assessment of the current situation. The major purpose of this study is to assess the confidence of tertiary care hospital interns and post-graduates in their communication skills.

Materials and methods

In May and June 2021, a cross-sectional survey was conducted among all interns and post-graduates from various departments at a tertiary care hospital and research center in Trichy, Tamil Nadu, India. The questions were designed to measure the confidence of physicians' communication with patients. The Likert scale was used to rate the "confidence in using" and "actual use" of communication skills with patients, which constitute two sections in our questionnaire. Google forms were used to collect data. The information was then exported to a Microsoft Excel spreadsheet. SPSS software version 22.0 was used to analyze the data.

Results

For confidence in their communication skills, the participants obtained a mean score of 2.98 (S.D. = 0.44). Participants obtained an average score of 2.28 (S.D. = 0.89) for practicing confident communication with patients. It was found that persons who had higher confidence levels had a negative correlation (ρ = -0.318) with that during their communication with patients and this was statistically significant (p-value = < 0.001)

Conclusion

Despite having confident communication skills, medical practitioners in India hardly ever practice them with their patients. This gap must be explored by conducting qualitative studies to address effective communication skills among health professionals.

## Introduction

One of the most essential aspects of medicine is the doctor-patient relationship. Many determinants influence this link, including socio-cultural patterns, economic levels, political systems, and health systems. "Trust" and "communication" are crucial tools for improving patient care and satisfaction. Patients may feel anxiety and peril if their caretakers lack or are unable to practice communication skills effectively [1].

The medical profession was once known for the "three Ts": talk, touch, and treat. A sympathetic conversation and a compassionate touch are all that is required for a patient to be treated for the sickness. Over time, two additional "T"s were added, test and technology, removing the most crucial "talk" and "touch." To provide safe, high-quality, and effective patient care, good communication skills are essential. A significant body of literature supports the relevance of students' understanding of communication skills [2,3]. This study also exemplifies how behaviors gained in communication skills training transcend the clinical situation and how much training is known to have long-term impacts on students' behavior.

In India, there is an increase in violence toward physicians. The news occasionally contains stories about a doctor who has been mistreated by his/her patients or their family. It was mainly due to dissatisfaction from the patient side [4]. The patient will be satisfied only if the physician builds empathy for the patient, exhibits genuine concern for the patient, and includes the patient in decision-making (patient-centered care). This can only be accomplished through the physician's exceptional communication abilities. It is a "need of the hour" to teach undergraduates effective communication skills, which are lacking in the medical curriculum [5].

Considering the amount and range of outside studies supporting the significance of communication skills, the lack of communication skills in the curriculum of Indian medical students is a significant accusation against the country's current medical education. Concerns regarding the absence of good doctor-patient communication among Indian doctors spurred an investigation of the current situation [6]. The objective of this study is to assess interns' and post-graduates' confident communication skills and its practice with patients in a tertiary care hospital.

## Materials and methods

Study design and setting

A cross-sectional study was carried out in a government tertiary care hospital and research center in Trichy, Tamil Nadu, India.

Study subjects

All medical interns and post-graduate professionals from all departments who agreed to participate in the study and give their informed consent were enrolled.

Sampling method and sample size

We used the "universal sampling method" and hence we were supposed to include all the interns and post-graduates of our government tertiary care center (n=215). Assuming a 10% non-response rate, the minimum sample size has to be 194 in this study.

Study period and study tool 

The study was carried out in May and June 2021. The questionnaire included questions designed to assess physicians' confidence in communicating with patients; it was developed by Ashbury et al. in 2001 and revised by a study conducted by an Iranian university in 2014 [7]. Based on the pilot study, the questions were slightly modified so that the participants could understand them. It is divided into two parts: "having confidence communication skills" and "actual use of confident communication skills with patients." The "having confidence communication skills" component (11 questions) was scored using a 4-point Likert scale and was operationally coded as "very low" for score 1, "low" for score 2, "high" for score 3, and "very high" for score 4. Similarly, a 5-point Likert scale was used to score the "actual use of confident communication skills with patients" (10 questions) and was coded as "very rarely" (< 20%), "rarely"(21-40%), "occasionally"(41-60%), "frequently"(61-80%), and "more frequently"(81-100%) for scores of 1 to 5, respectively.

 Data collection method

The data were collected online using Google forms. Individually, a Google Form link was sent to all interns and post-graduates through Gmail and WhatsApp. Those who did not respond after three attempts were considered non-responders. The results recorded in a Google Spreadsheet were statistically analyzed.

Statistical analysis

SPSS software Version 22.0 (IBM Corp., Armonk, NY) was used to analyze the data. Frequencies and percentages were used to characterize the study group's characteristics and various responses on the Likert scale. For both the individual and overall items, the mean scores (SD) were computed. The relationship between the two dimensions of confident communication skills was quantitatively linked using Spearman's rank correlation test (ρ).

Ethical clearance 

Ethical clearance was obtained from the Institutional Ethics Committee in Trichy, Tamil Nadu, India (I.E.C. No. 46/2020).

## Results

Among the total 215 study subjects in the government tertiary hospital, 200 participated in this study, and the non-response rate was 7%.

Table 1 describes the socio-demographic variables of the study participants. In our study, 83% of participants were between the ages of 20 and 25 years and 17% were between the ages of 26 and 40 years. In terms of gender, 55% of those who participated were female, while 45% were male. Of the study participants, 77% were interns, while 23% were post-graduates from departments such as microbiology, community medicine, radiology, surgery, medicine, pediatrics, dermatology, ophthalmology, psychiatry, and anesthesia. The majority of participants (65%) lived in cities, whereas 35% lived in rural regions.

**Table 1:** Socio-demographic variables and characteristics of the study population.

Characteristics	Categories	Frequency (n=200)	Percentage
Age	20-25 years	166	83%
	26-30 years	20	10%
	31-35 years	10	5%
	36-40 years	4	2%
Gender	Male	90	45%
	Female	110	55%
Intern/post-graduate	Intern	144	77%
	Post-graduate	56	23%
Permanent residence	Urban	130	65%
	Rural	70	35%

To assess participants' confidence in communication, those who agreed to participate were given a questionnaire with two sections: one on "confident communication competence" (11 questions) and one on "actual use of confident communication" (10 questions). Tables 2, 3 illustrate their Likert scale responses, respectively.

**Table 2:** Assessment of confidence in communication skills on a Likert scale ^a^Likert score (4 out of 4). ^b^Likert score (3 out of 4). ^c^Likert score (2 out of 4). ^d^Likert score (1 out of 4) SD, standard deviation

S. no.	Questions assessed	Mean (SD)	Level of confidence, n (%)			
			Very high^a ^	High^b^	Low^c^	Very low^d^
1.	Explaining treatment choices to your patient in a way that ensures your patient's understanding	3.15 (0.55)	134 (67%)	48 (24%)	18 (9%)	0 (0%)
2.	Explaining the present medical situation to him/her in a way that allows him/her to cope with his/her concerns	3.14 (0.61)	120 (60%)	54 (27%)	26 (13%)	0 (0%)
3.	Actively engaging patients and explaining to them the potential advantages and hazards of the proposed tests, treatments, and treatment choices (including medication)	3.01 (0.64)	118 (59%)	42 (21%)	40 (20%)	0 (0%
4.	Offering your patient precise advice about choices available and addressing alternative methods to resolve a common health problem	2.97 (0.74)	96 (48%)	50 (25%)	52 (26%)	2 (1%)
5.	Showing empathy to your patient about his/her dilemma	2.85 (0.83)	94 (47%)	44 (22%)	50 (25%)	12 (6%)
6.	Recognizing and responding to your patient's vocal expressions	3.01 (0.75)	104 (52%)	52 (26%)	38 (19%)	6 (3%)
7.	Recognizing and acting on your patient's non-verbal cues (facial expression, eye contact, gestures, body movement, and posture)	2.7 (0.8)	82 (41%)	34 (17%)	74 (37%)	10 (5%)
8.	Even if your patient is challenging, you can communicate successfully with him/her	2.91 (0.69)	108 (54%)	38 (19%)	52 (26%)	2 (1%)
9.	Establishing the patient's commitment to attempting to adhere to the treatment plan that you prepared with your patient	2.86 (0.69)	106 (53%)	34 (17%)	58 (29%)	2 (1%)
10.	Using the final few minutes of the meeting to summarize the key concerns covered	3.01 (0.74)	96 (48%)	54 (27%)	48 (24%)	2 (1%)
11	Establishing and sustaining trust	3.25 (0.65)	102 (51%)	74 (37%)	12%)	0 (0%)

**Table 3:** Assessment of confidence in communication during practice with patients. ^a^Likert score (5 out of 5). ^b^Likert score (4 out of 5). ^c^Likert score (3 out of 5). ^d^Likert score (2 out of 5). ^e^Likert score (1 out of 5)

S. no.	Questions assessed	Mean (SD)	Confidence in communication during practice with patients, n (%)				
			More frequently^a^	Frequently^b^	Occasionally^c^	Rarely^d^	Very rarely^e^
1	You addressed your patient politely, warmly, and cordially	2.1 (1.14)	12 (6%)	12 (6%)	34 (17%)	68 (34%)	74 (37%)
2	Use open-ended inquiries to enable your patients to actively participate and share their difficulties	2.39 (1.1)	8 (4%)	28 (14%)	44 (22%)	74 (37%)	46 (23%)
3	You encouraged your patients in expressing their feelings and responding in a helpful manner	2.24 (1.18)	12 (6%)	18 (9%)	44 (22%)	58 (29%)	68 (34%)
4	You actively conveyed understanding and empathy for your patients' difficulties	2.38 (1.18)	12 (6%)	28 (14%)	36 (18%)	72 (36%)	52 (26%)
5	You tried to figure out your patients' psychological, emotional, and social issues	2.34 (1.13)	12 (6%)	18 (9%)	48 (24%)	70 (35%)	52 (26%)
6	You sought to educate your patients and ensured that they knew their health issues and their etiology	2.13 (1.14)	12 (6%)	12 (6%)	38 (19%)	66 (33%)	72 (36%)
7	Try to actively include the patient and then assess whether or not the patient has a reasonable understanding of the possible therapy alternatives	2.27 (1.06)	6 (3%)	22 (11%)	46 (23%)	72 (36%)	54 (27%)
8	Answered your patients' inquiries in as much detail as possible	2.19 (1.06)	8 (4%)	16 (8%)	40 (20%)	78 (39%)	58 (29%)
9	After developing a treatment plan, you attempted to get your patients' commitment to try to follow the treatment plan	2.46 (1.13)	14 (7%)	20 (10%)	52 (26%)	72 (36%)	42 (21%)
10	You effectively engaged your patients throughout the conversation and then summarized everything for them	2.32 (1.2)	16 (8%)	16 (8%)	42 (21%)	68 (34%)	58 (29%)

Table 2 describes the representation of responses to confident communication skills on a Likert scale of 4. For the majority of the items, the scores averaged above 3, indicating the presence of high to very high confidence levels among medical professionals. We witnessed that 91% of study participants were highly confident enough about explaining treatment options to the patient in a way that ensures the patient's high level of understanding, 88% were highly confident in establishing and sustaining trust among patients, 87% of the study participants felt highly confident about explaining the current medical problem to their patients in a way that helps them deal with their worries, and 80% of study participants were highly confident about actively involving patients and explaining the potential benefits and risks (including medication).

However, 42% of participants felt a lack of confidence in picking up non-verbal cues during patients interactions, 31% had lesser confidence in showing empathy in addressing the dilemma of patients, 30% had lesser confidence in establishing patients' commitment to treatment adherence, and 27% lacked competency in successfully communicating with challenging patients.

Table 3 describes the representation of responses to the actual use of confident communication skills on the Likert scale score of 5. The mean score for the majority of the items was found to be less than 2.5, indicating lower confidence while interacting with patients in reality. We noticed that 73% of participants were less likely to encourage the patients in expressing their feelings and giving a helpful response, 71% were less likely to speak in a polite, warm, and friendly manner, 69% hardly educated the patients and ensured their comprehension, 68% barely answered patients' questions in detail, and 63% engaged them throughout the conversation and summarized less effectively.

**Figure 1:**
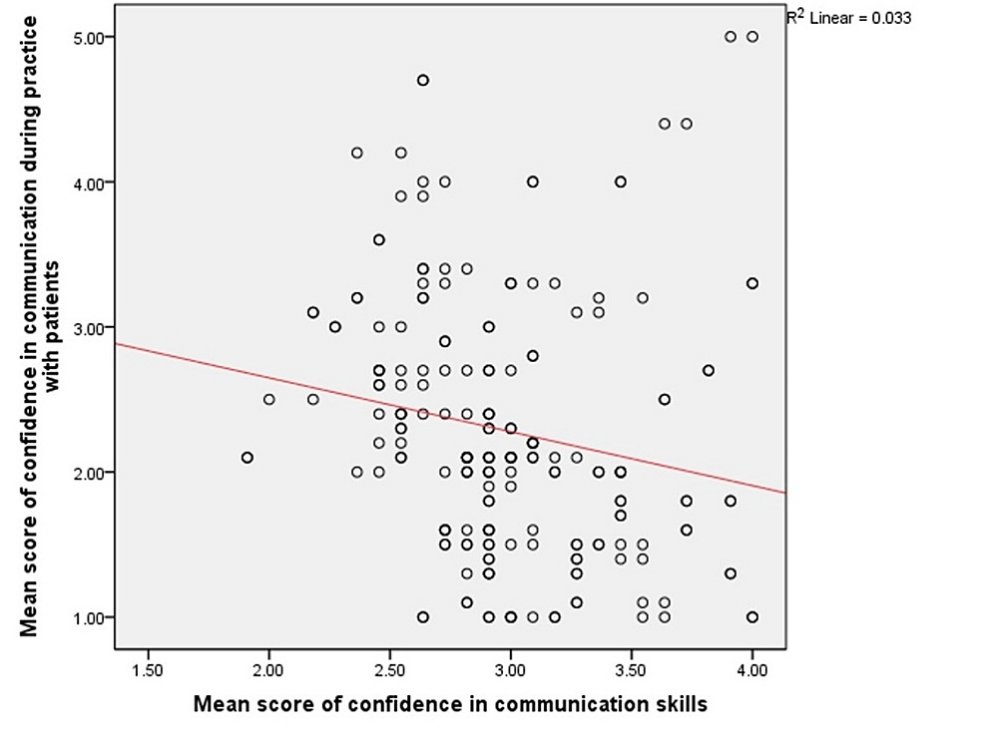
Scatter diagram showing a relationship between mean scores of confidence in communication with practice with patients. R^2^, correlation coefficient

For confidence in their communication abilities, the participants obtained an average score of 2.98 (SD = 0.44). Participants obtained an average score of 2.28 (S.D. = 0.89) for practicing confident communication with patients. Figure 1 shows the correlation between the overall mean scores for having confident communication skills and those used in practice with patients. It was found that doctors who presumed to have higher confidence in their communication skills were lacking it in practice as given by the negative correlation observed in Spearman's rank correlation test ( ρ = -0.318), with a statistically significant p-value of <0.001.

## Discussion

As doctors advance in their training, it has been observed that their communication skills diminish and that they lose interest in providing holistic patient care. Our study has highlighted the domains that need to be focused on during patient interaction for patient-centered care.

The doctors are presumed to have very high confidence in disciplines such as explaining treatment options to the patient in a way that ensures the patient's high level of understanding (67%), explaining the current medical problem to the patients in a manner that could alleviate their concern (60%), and actively involving patients and explaining the potential benefits and risks (59%). According to Jalalvandi et al.’s [8] study in Iran, 59% of study participants were confident in describing treatment alternatives to the patient in a way that assures the patient's high level of knowledge, 51.7% were confident in explaining the current medical condition to him/her in a way that helps him/her deal with his/her fears, 53.2% were confident in actively involving the patients, and 52% were confident in discussing the possibilities. In comparison to the previous study, both outcomes were consistent. Thus, around 60% of medical students worldwide are confident in their patient communication abilities. A lot of research papers suggest that both graduate physicians and interns have avoided engaging with patients. The majority of patients reported that their doctors did not care about their problems; they neither listened nor provided them with more information on the sickness [9].

The medical professionals in our study had very low confidence in diagnosing patients' non-verbal cues (37%), making them adherent to the treatment and following up on patients' treatment plans (29%), and coping with the challenging patients with skillful communication (26%). Only moderate levels of scores were observed in interns' capacity to communicate with patients in research by Farajzadeh et al. [10]. Some communication issues in the doctor-patient relationship may be caused by a lack of real information, as reported by Tavakol et al. in their study, which also found that knowledge constraints are the primary reason for poor communication among medical students [11].

We noticed that 73% of participants were less likely to encourage the patients in expressing their feelings and giving a helpful response, 71% were less likely to speak in a polite, warm, and friendly manner, 69% hardly educated the patients and ensured their comprehension, 68% barely answered patients' questions in detail, and 63% engaged them throughout the conversation and summarized less effectively.

The medical professionals practicing communication skills with patients in our study very rarely spoke in a polite, warm, and friendly manner (37%), very rarely educated and ensured the patient's understanding of health issues (36%), very rarely expressed concerns about patients' feelings and supported them with a helpful response (34%), very rarely addressed the patient's queries (29%), very rarely engaged them actively during the discussion, (29%) and very rarely summarized the material for them at the conclusion (29%).

According to the study conducted by Jalalvandi et al. [8] in Iran, 21.7% of respondents used confident communication skills to speak to the patient in a polite, warm, and friendly manner; 17.4% had to educate the patients and also make sure he/she understood their health problems and their etiology, 21.7% assisted the patients in expressing their feelings and responded in a supportive manner, and only 9% answered the patients' questions in detail, actively engaged them. In comparison to the previous study, current study participants used their communication skills with patients more effectively. The medical professionals in our study were found to practice better communication skills than that observed by Jalalvandi et al. [8].

The mean score of having confident communication skills was negatively correlated to that practiced with the patients. Therefore, doctors with higher confidence in communication abilities practice them infrequently. In contrast, Jalalvandi et al. discovered a significant and positive association between interns' confidence and communication abilities in their study [8]. According to Morgan and Cleave-Hogg, there is no clear link between having confidence and actually applying confident communication abilities [9]. Hence, there is an unacknowledged gap between comprehending and adopting communication techniques by medical professionals. This might be due to interference from the differences in individual personality traits, emotional intelligence, cultural diversity, and so on that hinder doctors' effective communication with patients and require further qualitative research in the future.

Limitations

The non-probability sample technique used to choose participants is one of the study's limitations. A subjective interpretation of the questionnaire will skew the study results because the data were collected online using a Google Form. Despite several attempts, we were unable to receive information from each and every health professional at the tertiary care hospital. This study was cross-sectional; longitudinal research on communication methods over time is needed in future studies to assess and make conclusions.

Strengths and recommendations

As our study is prone to social desirability bias, methods such as indirect questioning and forced choice options were applied to reduce them. We recommend structured training programs comprising personality development and techniques to improve communication skills be imbibed throughout the undergraduate and post-graduate medical curriculum in order to strengthen the doctor-patient relationship.

## Conclusions

Healthcare professionals in India were confident in their abilities to explain proposed investigations, therapies, and treatment options, but they lacked confidence in their capacity to recognize patients' non-verbal cues, show empathy for them, follow through on their advised course of treatment, and cope with challenging patients. Only a very few were approachable, kind, and pleasant in a way that allowed patients to express their feelings and obtain encouraging replies. Despite the belief that they have confident communication skills, medical practitioners hardly practice them with real patients. In order to address effective communication skills among health professionals, this disparity needs to be investigated through qualitative studies.
